# Should paramedics ever accept patients’ refusal of treatment or further assessment?

**DOI:** 10.1186/1472-6939-14-44

**Published:** 2013-11-04

**Authors:** Halvor Nordby

**Affiliations:** 1 Lillehammer University College, Faculty of Health and Social Work, 2604 Lillehammer, Norway

## Abstract

**Background:**

This case report discusses an ethical communication dilemma in prehospital patient interaction, involving a patient who was about to board a plane at a busy airport. The article argues that the situation raised dilemmas about communication, patient autonomy and paternalism. Paramedics should be able to find good solutions to these dilemmas, but they have not received much attention in the literature on prehospital ambulance work.

**Case presentation:**

The patient had chest pains that were consistent with serious heart disease, but she wanted to catch her plane and was unwilling to let paramedics assess her heart activity by means of an electrocardiogram (ECG). The paramedics had to decide, there and then, whether the patient’s refusal to submit to an ECG should be respected, or whether they should set the patient’s expressed wishes aside by exercising verbal power and persuasive communication techniques. The paramedics chose to do the latter. It later turned out that the patient was grateful that the paramedics had been very direct, almost brutal, in their communication. When the patient regained her autonomy, she saw clearly that taking time to obtain and monitor an ECG was the best option for her.

**Conclusion:**

Looking forward in time might be a good professional strategy for deciding whether ethical paternalism in communication is justified. If there is good reason to believe that patients who later regain their autonomy will agree that paternalistic verbal actions were in their best interests, and if acting in accordance with patients’ preferences can have severe negative health consequences for them, then paramedics have good reason to believe that ethical paternalism is justified.

## Background and case presentation

The case is typical of what paramedics sometimes call 'airport situations’: it is not uncommon that people who are about to travel by plane experience stress and fatigue. Such psychological factors can be the extra element that triggers chest pains and possible underlying heart disease. The case concerned a situation of this kind involving a young woman who was about to board a plane.

At the departure gate the patient experienced severe chest pains. Airport crew summoned paramedics. When the paramedics arrived the patient was pale and sweating and complained of pain. She was nevertheless unwilling to let them carry out the necessary activities for obtaining an ECG (the paramedics had standard equipment that provided 12-lead monitoring). The reason she gave was that she had to catch a plane that was leaving in 45 minutes. She told the paramedics that she was travelling to a business meeting that it was imperative for her to attend, that her job and career were at stake. She also told the paramedics that there were no other planes leaving later that day that went to the city she was travelling to, and that many people were waiting for her. These people had travelled from all over the country just for this meeting today, and she said, 'I absolutely must catch this plane.’ She promised the paramedics that she would seek medical advice in the city she was going to later that day, right after the business meeting. She also said that the pain was not as bad as it had been when the airport crew dispatched the paramedics, and that she now felt better.

### The dilemma

The context of the patient encounter put extra pressure on the paramedics. The patient was stressed, they had to assess the patient in the middle of a busy airport, and the plane schedule made it imperative to think and act quickly. The patient could have a serious heart disease, so instant support to prevent possible dramatic consequences could be crucial. Nevertheless, the paramedics faced a dilemma that did not have an obvious answer: Was it ethically acceptable to let the patient board the plane, or was it morally justified to refuse to allow her to go? The latter course of action would involve ethical paternalism [1] – a decision to overrule another person’s preferences by not allowing the person to act in accordance with her own expressed wishes [1-3].

Initially, neither of these two options seemed to be a good ethical solution to the dilemma. Letting the patient travel by plane for three hours without any chance of proper medical treatment on board could involve great danger for her. Furthermore, the paramedics were aware that they had a professional duty to consider the potential consequences and safety of the other people on the plane and assess whether the patient’s state of illness could have negative consequences for them and their journey. But forcing the patient to submit to an examination and refusing to let her go fell outside the paramedics’ formal authorization and power of attorney. They were not entitled to exercise physical force by stopping the woman from attempting to board the plane.

The paramedics chose a better, third alternative: Within the time span they had at their disposal, they attempted to persuade the patient to accept that it was crucial for her to submit to an ECG, by explaining in more detail that she might have a significant heart problem and that this problem could be very serious. They informed her about the ECG procedure and told her how this could reveal abnormal heart function. However, this did not help. Even though the paramedics communicated facts and information about possible causes of her pain and the technical nature of their equipment, the patient insisted that she 'had to go’.

It was at this stage that the paramedics chose to be much more direct in their communication. They told the patient that there was a 'significant risk’ that the pain she had experienced '*could* be caused by a very serious heart disease.’ Furthermore, in the dialogue with the patient it had become clear that the woman was the mother of two young children. One of the paramedics asked her: 'Is your career more important to you than your own children. Are you sure that you are willing to risk dying and leaving them as orphans?’ This made a crucial difference. The patient saw the situation in a new light. She consented to submit to an ECG and said that she would cancel the meeting and not take the plane. She was not happy about this, but she nevertheless deferred in action. She chose this option of her own free will.

### The patient’s autonomous perspective

A significant aspect of the case is that the patient subsequently agreed that the very direct communication style had been justified. Two months later the patient got in touch with the ambulance station at the airport where the paramedics worked. She sent an email to the station manager, asking him to forward it to the paramedic crew who had taken care of her. In this correspondence she expressed gratitude to the paramedics for having made her change her mind about the importance of the medical examinations.

Part of the email reads as follows: 'There and then I had lost touch with myself. I was stressed, tired and focused solely on the meeting. When I regained a more sober perspective, I understood very well that it was correct to let them do their work.’

The patient also wrote that further medical examination could *not,* in fact, document any underlying heart problem, but she understood that there and then, at the airport, the possibility of serious disease could not be ruled out. She realized that the paramedics had to take this possibility into consideration. She now wanted them to know that she was very grateful that they had been so direct in their communication with her.

### Context

The patient was contacted through the ambulance station she had been in touch with. She was asked for permission to use the encounter, described in anonymous terms, in a case report of the present kind.

The patient was given all relevant information and the opportunity to ask questions about the writing project. The patient responded right away that she thought it was a very good idea to write an article about the situation, in the light of the fact that she was very grateful to the paramedics for what they had done. She wrote: 'If you write about the situation, it will hopefully give other paramedics the confidence to put the same kind of pressure on patients in similar circumstances. It could also help other patients who have lost touch with reality’.

The above account of the case study is based on the paramedics’ narrative of the patient encounter, but it has been transcribed in completely general terms so that it is not connected, and cannot be traced, to any specific person, place or circumstances. By specifying that the case is an example of a particular *type* of case, it is easier to elucidate its general significance. That is, the analyses below apply to a variety of cases that are more or less similar to situations of the kind described above. The arguments are relevant in many different contexts involving patients whose ability to make autonomous decisions is in doubt, not only 'airport situations’ of the kind described above. It should be fairly easy for the reader to understand the generalization value of the main arguments.

#### *Analysis*

The case initially seemed to involve a choice between two courses of action: letting the patient decide or forcing her to submit to a medical assessment. Both alternatives were perceived as having problematic aspects, and that was the main reason why the situation appeared to be so challenging for the paramedics.

The task of choosing the right course of action in situations of the above kind can be traced to a fundamental tension between two aspects of the professional ethical obligations paramedics have. On the one hand, they have a duty to respect autonomous wishes and involve patients in decision processes, especially when patients make it clear that they want to be involved. On the other, paramedics also have a fundamental responsibility to prevent harm and serious negative health consequences for patients. This tension involves a conflict between two corresponding ethical principles. Respecting autonomous wishes corresponds to the idea that patients should be allowed to live their lives in the way they want. Preventing harm corresponds to the idea that professional paternalism is sometimes justified in order to secure necessary assessment and treatment.

The above case presented itself as a dilemma of choosing between these two principles. The most striking aspect of the paramedics’ actions is that they avoided this 'either-or’ dilemma by pursuing a 'middle course’ that seemed more promising as an ethically acceptable solution: they used communication to try to persuade the patient to change her mind.

In general, such communication should ideally be *neutral*. When challenging a patient’s preferences, health workers should start out by attempting to give a balanced and informative account of their medical perspective on the patient’s symptoms and possible causes [4,5]. By communicating professional knowledge it is often possible to give patients a new perspective on their illness and states of ill health – a perspective that can lead them to revise their wishes [2,6,7]. The paramedics clearly acknowledged this when they started out by giving the patient explanations of the possible causes of the pain she experienced and the importance of obtaining an ECG.

The purpose of this kind of informative communication is to convey knowledge that can contribute to *informed* patient preferences. As Young [8], 442 notes, this means that the patient “must be competent, must understand the information disclosed to her and must give (or withhold) her consent freely.” For a patient to give informed consent (or informed refusal of treatment), the patient must be able to make autonomous choices:

…when a patient exercises her autonomy she decides which of the options for dealing with her health-care problem (including having no treatment at all) will be best for her, given her particular values, concerns and goals. A patient who makes autonomous choices about her health care is able to opt for what she considers will be best for her, all things considered [ibid].

In the present case, the fundamental problem was that it was far from clear that the patient, even after the initial dialogue about possible causes, 'exercised her autonomy’ when she continued to be unwilling to defer. The patient was given relevant factual information, but she still refused to let the paramedics obtain an ECG. They continued to probe her understanding in order to assess her autonomy, but she did not change her mind. The alternative the paramedics then chose was to put pressure on her by saying, in very direct terms, that her life was more important, both to her and her children, than one business meeting.

Ordinarily, the ethical status of this kind of communicative pressure is ethically questionable (4). Using persuasion techniques for the purpose of changing patient preferences involves the exercise of verbal power, and such use of professional power requires a special ethical justification. Persuasive communication techniques and brutal communication, communication that is very direct, perhaps even commando-like - should not generally be used as everyday tools for challenging patient preferences. When health workers use verbal power in a given situation, it is imperative that they are able to explain why the situation entitles them to do so, why they are justified in overruling the norm that patient interaction should not involve the use of any kind of power. Appealing to the idea that speech acts are 'merely’ verbal and not non-verbal, physical actions is not sufficient. Verbal actions are actions just like physical actions, and should therefore also be evaluated ethically.

### The significance of doubt

As the paramedics in the above case implicitly understood, doubts about autonomy and negative consequences of patient preferences can jointly constitute sufficient reason for not conforming to the principle that provider-patient communication should be neutral: if it is reasonable to believe that patients are not fully autonomous, and if letting them decide can have serious negative consequences for them, then health personnel may be entitled to use persuasion techniques that go beyond pure factual and informative communication. The paramedics were clearly aware of this. They understood that the *intrinsic* negative aspects of using persuasion techniques have to be balanced against other ethical considerations. Sometimes other aspects of a situation, such as the need to prevent possible serious consequences for a patient, outweigh the negative aspects of using persuasion techniques. Furthermore, using such techniques is not synonymous with failing to respect a patient’s autonomy. Obviously, giving patients neutral information helps them to develop autonomous preferences. But even when the communication is far from neutral, the communication can sometimes be consistent with the wishes patients would have had if they had been more autonomous. So in some cases this kind of communication can also, in a more subtle sense, be understood as communication that respects autonomy. This is precisely what happened above: the paramedics thought that the direct communication, there and then, was justified as a means of achieving a result that was in the patient’s best overall interests.

It is also important to note that in order for persuasion techniques to be ethically acceptable in a given situation, health personnel do not have to know for certain that patients have lost their capacity to make autonomous decisions. Thus, in the above example the paramedics did not have to know for certain that the patient’s preferences were not based on rational reasoning. This is an important point, as there are many cases that fall into a grey zone, where it is difficult to decide whether patients are capable of making autonomous choices and giving informed consent [9,10].

The above case illustrates that there are many ways patients might *appear* to lose their autonomy. As Young [8], 442 notes, “The effects of injury, illness or medication can increase the probability that a patient will make choices that appear unbalanced and so call into question her competence to make decisions about her health care.” But knowing that states of ill health *can* increase the probability of unbalanced decisions is not the same as knowing that they actually do so in a given case. In many areas of medical practice it is often difficult to determine whether a patient’s wishes are sufficiently autonomous, and prehospital work is definitely such an area.

However, in the above case there was clearly sufficient doubt. Furthermore, the case illustrates that loss of autonomy is not necessarily grounded in impaired mental capacities due to documented injury or disease. The problem was 'merely’ that the patient was stressed and in a hurry. She had a narrow psychological focus on the business meeting – a focus she later regretted that she had.

At the same time it is important to remember that mere doubt about autonomy is insufficient as an ethical justification for exercising power through persuasion techniques. If letting a patient decide clearly has no substantial negative consequences, then health workers should focus on neutral communication, even when they have reason to doubt that the patient is autonomous. But this was not the case above or in other relevantly similar situations. There was (i) significant doubt about the patient’s autonomy, but also (ii) doubt about serious negative consequences of letting the patient decide. *Both* of these conditions were met, this was something the paramedics clearly recognized, and that is why they were entitled to put communicative pressure on the patient.

As described above, the patient later expressed her gratitude to the paramedics for being so direct in their communication with her. Obviously, there and then at the airport, the paramedics could not know for certain how she would later evaluate their verbal actions. But this is not the crucial point. What is important is that the paramedics had good reason to believe that the patient’s preferences were not fully autonomous, and that there was a significant probability that she would, when she regained a more sober perspective, agree that medical assessment was more important than the business meeting. In fact, even if she had not changed her mind about this, the paramedics would have been entitled to put pressure on her. It was not what happened afterwards that made them entitled to do so. It was the context at the airport and the limited knowledge about the patient they possessed there and then that gave them good reason to put pressure on her.

This is an important point, since some might think that the paramedics’ judgements were based on speculations about presumptive autonomous consent. But that was not the case. The paramedics acted, and they had to act, on the basis of the observations and understanding they had. This gave them good reason to deviate from the principle of neutral communication.

The same point would, in fact, be valid if the patient had not consented to medical assessment at the airport. As it turned out, using direct communication techniques was the key to finding a solution to the conflict: the patient deferred, although somewhat unwillingly. But we can imagine another possible outcome. What if the patient had not deferred? What should the paramedics have done? These questions raise further ethical issues that cannot be addressed within the limits of this article, but one point about this possibility should be mentioned: The aim here has been to argue that the doubt about autonomy and possible serious consequences gave the paramedics good reason to communicate as they did. Again, there and then, when they chose to use persuasion techniques, they could not know for sure how the patient would respond. But the limited knowledge they had when they had to make a choice gave them *sufficient* reason to act paternalistically.

It should also be emphasized, as a final general point c that this idea about sufficient reason is consistent with holding that paramedics are sometimes entitled to communicate paternalistically for other reasons than doubt about patient autonomy. What the above case so strikingly illustrates is that such doubt is one legitimate source of verbal paternalism in cases where acting in accordance with patients’ expressed preferences can have serious negative consequences for them.

## Conclusions

There are many ways of addressing issues of ethical paternalism in cases where patients are doubtfully capable of making autonomous decisions and not, apparently, sufficiently focused on possible negative consequences of their own preferences. One strategy is to try to look forward in time. When the patient regains his or her autonomy – or when there is less doubt about loss of autonomy - will he or she look back and be glad that the health workers acted paternalistically? This is not the only possible way of considering the justification of paternalism, but it can be one useful and legitimate strategy. In other words, the crucial point is that paramedics should be able to provide good reasons for their ethical decisions, and that the 'looking forward in time’ strategy can give them such reasons.

In the above case the patient later said that she was grateful that the paramedics put communicative pressure on her, which is why the case is such a good illustration of how the strategy can be employed. By thinking about what patients will say when they have had time to calm down and reflect on their situation and relevant choices, health workers can transcend the perspectives patients have in the situation: It is, from a professional perspective, possible to arrive at more substantial ethical conclusions about patients’ fundamental perspectives on themselves, different actions and their own best interests.

As emphasized above, it is difficult to know for sure how patients will evaluate actions later on. But again, reasonable doubt about autonomy and possible serious consequences there and then is all that is required. If there is *sound* reason to believe that patients might change their minds later when they have more informed and rational perspectives, then health workers have corresponding good reason to use persuasion techniques to prevent negative consequences. It is better to be on the safe side and prevent possible serious consequences than to accept wishes that may not be autonomous.

## Consent

Written informed consent was obtained from the health workers and the patient for publication of this case report. All names and descriptions have been formulated in anonymous terms. No part of this case report can be traced to an actual place or event.

## Competing interests

The author declares that he has no competing interests.

## Authors’ information

HN received a D.Phil in Philosophy of Mind and Language at the University of Oxford in 2001, and is now working as a Professor at the University of Oslo, Faculty of Medicine, and at the University College of Lillehammer, Faculty of Health and Social Sciences. His main research interests include health management, provider patient communication and ethics in health care. In his research he has focused extensively on communicative challenges and ethical dilemmas in ambulance services, and he has, for many years, worked closely with medical rescue teams and the national ambulance services in Norway. He has written many books and articles on issues related to medical emergency services, health management, ethics and communication.

## Pre-publication history

The pre-publication history for this paper can be accessed here:

http://www.biomedcentral.com/1472-6939/14/44/prepub
